# The centromeric nucleosome-like CENP–T–W–S–X complex induces positive supercoils into DNA

**DOI:** 10.1093/nar/gkt1124

**Published:** 2013-11-14

**Authors:** Kozo Takeuchi, Tatsuya Nishino, Kouta Mayanagi, Naoki Horikoshi, Akihisa Osakabe, Hiroaki Tachiwana, Tetsuya Hori, Hitoshi Kurumizaka, Tatsuo Fukagawa

**Affiliations:** ^1^Department of Molecular Genetics, National Institute of Genetics, Mishima, Shizuoka 411-8540, Japan, ^2^The Graduate University for Advanced Studies (SOKENDAI), Mishima, Shizuoka 411-8540, Japan, ^3^Medical Institute of Bioregulation, Kyushu University, Fukuoka, Fukuoka 812-8581, Japan, ^4^JST, PRESTO, Fukuoka 812-8582, Japan and ^5^Laboratory of Structural Biology, Graduate School of Advanced Science and Engineering, Waseda University, Shinjuku-ku, Tokyo 162-8480, Japan

## Abstract

The centromere is a specific genomic region upon which the kinetochore is formed to attach to spindle microtubules for faithful chromosome segregation. To distinguish this chromosomal region from other genomic loci, the centromere contains a specific chromatin structure including specialized nucleosomes containing the histone H3 variant CENP–A. In addition to CENP–A nucleosomes, we have found that centromeres contain a nucleosome-like structure comprised of the histone-fold CENP–T–W–S–X complex. However, it is unclear how the CENP–T–W–S–X complex associates with centromere chromatin. Here, we demonstrate that the CENP–T–W–S–X complex binds preferentially to ∼100 bp of linker DNA rather than nucleosome-bound DNA. In addition, we find that the CENP–T–W–S–X complex primarily binds to DNA as a (CENP–T–W–S–X)_2_ structure. Interestingly, in contrast to canonical nucleosomes that negatively supercoil DNA, the CENP–T–W–S–X complex induces positive DNA supercoils. We found that the DNA-binding regions in CENP–T or CENP–W, but not CENP–S or CENP–X, are required for this positive supercoiling activity and the kinetochore targeting of the CENP–T–W–S–X complex. In summary, our work reveals the structural features and properties of the CENP–T–W–S–X complex for its localization to centromeres.

## INTRODUCTION

The kinetochore is assembled on centromere regions to provide an essential structure for faithful chromosome segregation (1,2). Although specific DNA sequences are dispensable for centromere function in vertebrates (3), the centromere contains a specialized chromatin structure that is distinct from other genomic loci. A critical feature of centromere regions is the presence of specialized nucleosomes in which histone H3 is replaced by the centromere-specific histone H3 variant CENP–A (2,4). CENP–A-containing nucleosomes exist at all functional centromeres, including neocentromeres (3,5,6), and ectopic targeting of CENP–A to a non-centromere region can induce kinetochore formation in diverse organisms (7–9).

Although CENP–A is a key component of centromere chromatin, additional factors are also involved in the formation of the specific centromeric chromatin structure. CENP–T, –W, –S and –X form a centromere-specific DNA-binding complex (10–12). The centromere localization of the CENP–T–W–S–X complex depends on CENP–A, but the CENP–T–W–S–X complex does not directly associate with CENP–A nucleosomes (10). This suggests that CENP–A and the CENP–T–W–S–X complex are distinct, but function coordinately to establish a centromere-specific chromatin structure (10). Despite the fact that the CENP–T–W–S–X complex is distinct from canonical histones at a primary sequence level, we demonstrated previously that the CENP–T–W–S–X heterotetramer is structurally homologous to the histone tetramer within the nucleosome (12). In addition, the CENP–T–W–S–X complex induces supercoils into DNA (12), suggesting that this complex bends DNA around its surface to form a nucleosome-like structure (13).

There is an active debate on the topology of nucleosomes at centromere regions (14). *In vitro* reconstituted human CENP–A nucleosomes are octameric and induce negative supercoils into DNA similar to canonical nucleosomes (15,16). In contrast, CENP–A nucleosomes isolated from *Drosophila* cells under some purification conditions are tetrameric and induce positive supercoils into DNA (17,18). Experiments with budding yeast minichromosomes also suggest that centromeric chromatin induces positive supercoils into DNA (18). Although it is still unclear if and why centromeric nucleosomes induce positive supercoils, the existence of nucleosomes with opposite topologies to those of canonical nucleosomes may function to mark this specialized genome region.

As recombinant vertebrate CENP–A and other histones that form octameric nucleosomes induce negative supercoils, if centromeric regions display positive supercoiling, other factors may contribute to this centromere topology. The histone-like CENP–T–W–S–X complex is a good candidate to function in establishing this specific centromere topology. Here, we characterized the DNA-binding properties of the CENP–T–W–S–X complex. We found that the CENP–T–W–S–X complex binds to ∼100-bp DNA primarily as a (CENP–T–W–S–X)_2_ structure and induces positive supercoils into DNA. We also found that the DNA-binding regions in CENP–T or CENP–W, but not CENP–S or CENP–X, are responsible for the positive supercoiling activity. Our work reveals the structural features and properties of the CENP–T–W–S–X complex for its localization to centromeres.

## MATERIALS AND METHODS

### DT40 culture and immunofluorescence

Chicken DT40 cells were cultured in Dulbecco’s modified medium supplemented with 10% fetal calf serum, 1% chicken serum, 10 μM 2-Mercaptoethanol, penicillin and streptomycin at 38.5°C in an atmosphere of 5% CO_2_. GFP–CENP–TΔC–CENP–S and FLAG–CENP–T histone-fold constructs under control of a CMV promoter were transfected into CENP–T conditional knockout DT40 cells (CT44–23) (10).

For immunofluorescence, DT40 cells were collected onto glass slides using a Cytospin 3 (SHANDON) and were fixed in 3% paraformaldehyde in PBS for 10 min at room temperature. The fixed cells were permeabilized in 0.5% NP-40 in PBS for 10 min at room temperature. Then the cells were incubated with primary antibody diluted with 0.5% BSA in PBS for 1–3 h at 37°C. Rabbit polyclonal antibodies against recombinant chicken Ndc80 (19) and CENP–T (10) were used. To detect FLAG-tagged proteins, mouse monoclonal anti-FLAG M2 antibody (Sigma) was used. After washing samples, the cells were incubated with FITC or Cy3 conjugated secondary antibodies (Jackson ImmunoResearch) diluted with 0.5% BSA in PBS for 30–60 min at 37°C. Chromosomes were counterstained with 4, 6-diamidino-2-phenylindole (DAPI). Immunofluorescence images were collected with a cooled EM CCD camera (QuantEM, Roper Scientific) mounted on an Olympus IX71 inverted microscope with a 100× objective lens together with a filter wheel and DSU confocal system. Z-section images were collected at 0.2 or 0.3 μm intervals and analyzed using MetaMorph software (Molecular Device).

### Protein purification

The chicken CENP–T–W, CENP–S–X, and CENP–T–W–S–X complexes were expressed and purified as described previously (12). MBP–CENP–T was prepared using a fusion of chicken CENP–T (amino acids 531–639) to MBP with a TEV cleavage site inserted between the two genes. MBP–CENP–T–CENP–W was co-expressed in bacteria and purified by amylose resin and gel filtration.

### Di-nucleosome preparation

Recombinant human histones H2A, H2B, H3.1, H4 and CENP–A were expressed and purified as described previously (16,20). To reconstitute H3-containing or CENP–A-containing octamers, equimolar amounts of each histone were dissolved in refolding buffer [20 mM Tris–HCl (pH 7.5), 7 M guanidine hydrochloride and 20 mM 2-Mercaptoethanol]. The samples were dialyzed against 2 M NaCl buffer [10 mM Tris–HCl (pH 7.5), 2 M NaCl, 1 mM EDTA and 5 mM 2-Mercaptoethanol] at 4°C and purified using a Superdex 200 column (GE Healthcare Biosciences). The purified histone octamer and DNA were mixed in a buffer containing 2 M KCl and mono-nucleosome were reconstituted by a salt dialysis method (16). The reconstituted mono-nucleosome was further purified by native polyacrylamide gel electrophoresis using a Prep Cell apparatus (Bio-Rad).

To prepare di-nucleosomes, we ligated two types of single mono-nucleosomes. The DNA used in mono-nucleosome reconstitution includes 145 bp of human α-satellite sequence and 11 bp additional linker sequence with an extra 3-base overhang. To prepare di-nucleosomes with a 25-bp linker, two types of mono-nucleosomes that have different linker sequences (3-base overhang 5′-GGA-3′ or 5′-TCC-3′) were prepared. These mono-nucleosomes were mixed and ligated by T4 DNA ligase for 2 days at 16°C. Ligated di-nucleosomes were purified by native polyacrylamide gel electrophoresis, using a Prep Cell apparatus (Bio-Rad). To prepare di-nucleosomes with a 100-bp linker, a 72-bp linker DNA derived from the pUC119 vector sequence containing an extra 3-base overhang (5′-TCC-3′ and 5′-CAA-3′) was generated. Ligation and purification were performed as described above.

### Gel shift assay

Gel shift assays were performed as described previously (12). For an assay with di-nucleosome and the CENP–T–W–S–X complex, 0.1 µM di-nucleosome and recombinant CENP–T–W–S–X complex were used in the buffer containing 10 mM Tris–HCl (pH 7.5), 100 mM NaCl, 2 mM 2-Mercaptoethanol. After incubation for 15 min at 37°C, the mixture was electrophoresed on a 4% native polyacrylamide gel and stained with ethidium bromide. For gel shift assays using naked DNA, 100 bp DNAs containing various sequence were amplified PCR using the following primers. A pUC119 DNA: pUC119-100 bp-Fw 5′-AACGACGAGCGTGACACCACGATGCCTGTA-3′ and pUC119-Rv 5′-TTAATTGTTGCCGGGAAGCTAGAGTAAGT-3′. An α-satellite DNA: alpha-sat-100 bp-Fw 5′-AATCTGCAAGTGGATATTTGG-3′ and alpha-sat-100 bp-Rv 5′-GCACAAAGAAGTTACTGAG-3′. A 601 positioning DNA: TN721-Fw 5′-ATCAGAATCCCGGTGCCGAGGCCGCTC-3′ and TN724-Rv 5′-CCCTTGGCGGTTAAAACGCGGGGGACAGCG-3′. A chicken centromere DNA: ggCEN-100 bp-Fw 5′-AAGCTGTCATATTGTCGGGAGAGAG-3′ and 1RU1.8 kb-#6-Rv 5′-CTTCTCCCCAGACTAGGACAATCTCCTC-3′. After incubation for 15 min at 37°C, the mixture of protein and DNA was electrophoresed on a 5%–20% gradient native polyacrylamide gel and stained with ethidium bromide.

### TEV digestion assay

The MBP-fusion tag is removable by Tobacco Etch Virus (TEV) protease. The MBP-fused protein–DNA complexes were digested with AcTEV protease (Invitrogen) in buffer containing 50 mM Tris–HCl (pH 7.5), 100 mM NaCl, 1 mM EDTA and 1 mM dithiothreitol (DTT) for 30 min at 30°C. Digested samples were electrophoresed on a 5%–20% native polyacrylamide gel and stained with ethidium bromide.

### Electron microscopy

Sample solutions were applied to copper grids supporting a continuous thin carbon film, incubated for 1 min, and then stained with three drops of 2% uranyl acetate. The specimens were examined with JEM 1010 electron microscope (JEOL), operated at an accelerating voltage of 100 kV. Images were taken with Bioscan CCD camera (GATAN). The step size of a pixel of the image was calibrated (1 pixel = 5.1 Å), using tobacco mosaic virus as a reference sample.

### Supercoiling assay

For the supercoiling assay, pBluescript containing ggCEN1 sequence (21) or φX174 RF I DNA was used. Relaxed plasmid DNA was prepared and the supercoiling assay was performed as described previously (12). Topoisomers were separated in 1% agarose gel in the presence or absence of chloroquine for 50 V overnight at 4°C and stained with ethidium bromide. We tested various concentrations of chloroquine and obtained best results at 1.2 µg/ml chloroquine, which was used for all experiments (see Supplementary Figure S2A). Two dimensional gel electrophoresis was performed as follows: in the first dimension, samples were separated in 1% agarose gel for 50 V overnight at 4°C in TAE buffer. The gel was then equilibrated for 2.5 h in TAE buffer containing 1.2 µg/ml chloroquine. The second dimension of electrophoresis was performed for 50 V 6 h at 4°C in the same buffer and gel was stained with ethidium bromide.

## RESULTS

### The histone-like structure of the CENP–T–W–S–X complex is an essential base for kinetochore assembly

**Figure 1.**
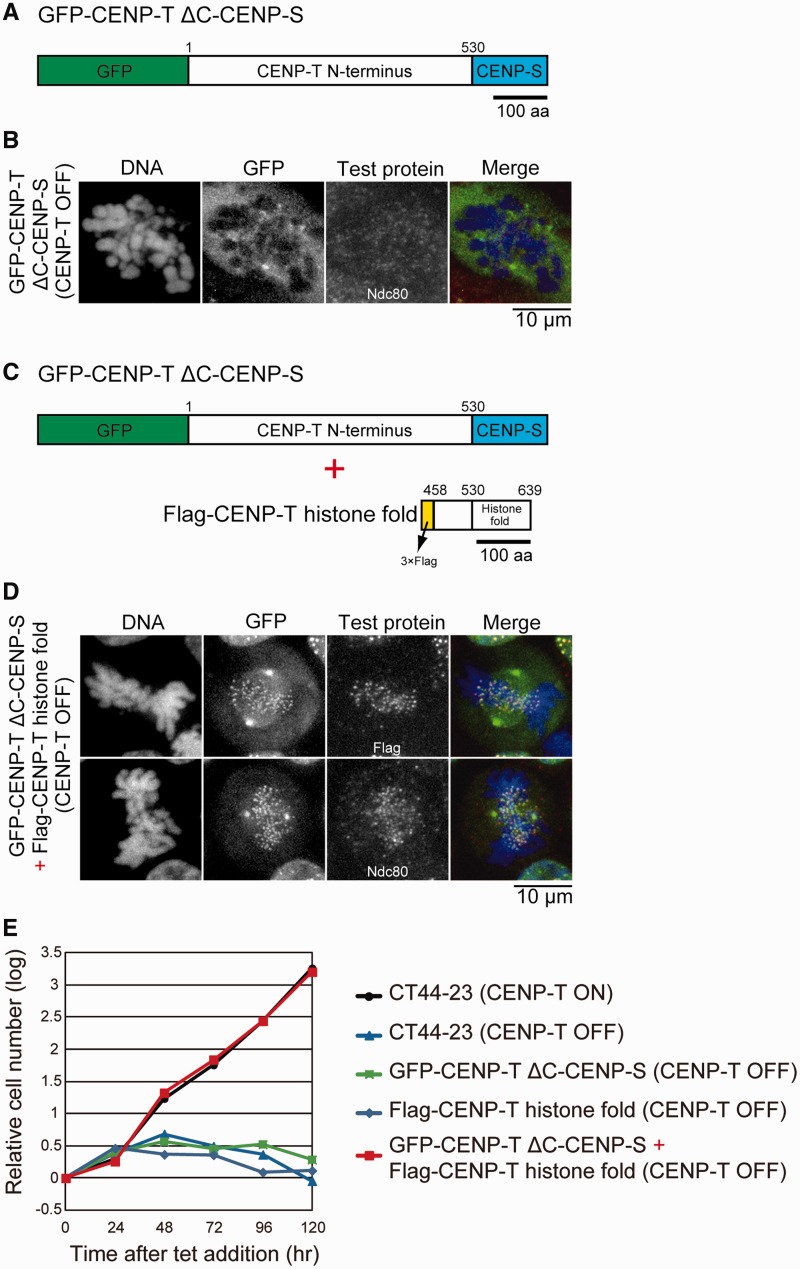
The histone-like structure of the CENP–T–W–S–X complex provides a platform for kinetochore assembly. (**A**) Diagram showing GFP (in N-terminal)/CENP–S (in C-terminal) double fusion protein with CENP–T N-terminus (1–530 aa) that lacks histone-fold region: GFP–CENP–TΔC–CENP–S. (**B**) Localization of Ndc80 in CENP–T-deficient cells expressing GFP–CENP–TΔC–CENP–S. (**C**) Co-expression of GFP–CENP–TΔC–CENP–S and the FLAG-tagged CENP–T histone fold (458–639 aa) region (FLAG–CENP–T histone fold). (**D**) Localization of Ndc80 in CENP–T-deficient cells co-expressing both GFP–CENP–TΔC–CENP–S and FLAG–CENP–T histone fold. (**E**) Growth curve of CT44-23 (CENP–T conditional knockout cells), CT44-23 expressing GFP–CENP–TΔC–CENP–S, CT44-23 expressing FLAG–CENP–T histone fold and CT44-23 co-expressing both GFP–CENP–TΔC–CENP–S and FLAG–CENP–T histone fold cell lines. CENP–T expression was conditionally controlled by tetracycline addition (CENP–T OFF) or the absence of tet (CENP–T ON) in CT44-23 cells.

We have shown previously that the histone-fold domain at the C-terminus of CENP–T is essential for its kinetochore localization, in part due to its interaction with the additional histone fold-containing proteins CENP–W, –S and –X (12,22). We have also demonstrated that the N-terminus of CENP–T plays a critical role in outer kinetochore assembly (10,22,23). To test the relationship between these two regions of CENP–T, we generated a construct in which the histone fold domain of chicken CENP–T (530–639 aa) was replaced with the histone fold domain from chicken CENP–S (CENP–TΔC–CENP–S; Figure 1A). This CENP–TΔC–CENP–S chimeric protein failed to target to kinetochores in the absence of endogenous CENP–T (Figure 1B). In cells expressing the CENP–TΔC–CENP–S chimera, the outer kinetochore protein Ndc80 also failed to localize to kinetochores, indicating that functional kinetochores did not form (Figure 1B). However, when we co-expressed the histone-fold domain from CENP–T (458–639 aa: lacking the N-terminal outer kinetochore assembly region) together with the CENP–TΔC–CENP–S chimera, we observed that the chimeric protein localized kinetochores (Figure 1C and D) through an interaction of the CENP–T histone fold with CENP–W and –X. In addition, we found that co-expression of these proteins fully rescued the CENP–T-deficient phenotype and kinetochore assembly (Figure 1E). These results indicate that the CENP–T–W–S–X complex provides two critical contributions to centromere function (centromere targeting and outer kinetochore assembly), but these are separable within the larger CENP–T–W–S–X complex. As the histone-fold region of the CENP–T–W–S–X complex is critical for its association with centromere chromatin, here we analyzed the DNA-binding properties of the histone-fold region of the CENP–T–W–S–X complex.

### The CENP–T–W–S–X complex binds preferentially to ∼100 bp linker DNA rather than nucleosomal DNA

The CENP–T–W–S–X complex associates with DNA, and therefore must assemble together with the other components of centromeric chromatin. However, the relationship between the CENP–T–W–S–X complex and the other nucleosomes present at centromeres is unknown. Therefore, we examined how the histone-like CENP–T–W–S–X complex associates with centromeric chromatin. Although the CENP–T–W–S–X complex requires CENP–A for its proper centromere localization, we have shown previously that CENP–T preferentially associates with histone H3, rather than CENP–A, at centromere regions based on biochemical co-purification experiments from human and chicken cells (10) and visualization of centromere chromatin using super resolution microscopy (24). Therefore, we tested the association of reconstituted CENP–T–W–S–X with either CENP–A- or H3-containing nucleosomes.

**Figure 2.**
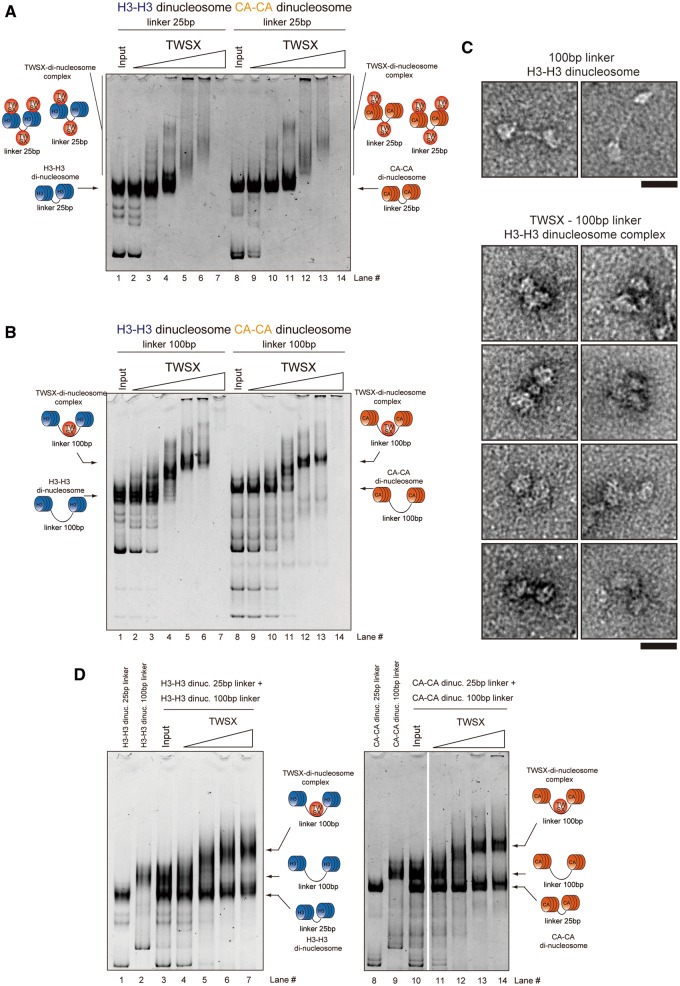
The CENP–T–W–S–X complex preferentially binds to ∼100 bp linker DNA rather than nucleosomal DNA. (**A**) Binding assays for the CENP–T–W–S–X complex to di-nucleosomes (0.1 µM) containing either CENP–A or histone H3 with 25 bp linker DNA. Different concentrations of the CENP–T–W–S–X complex (lane 2, 9 - 0.1 µM; lane 3, 10 - 0.2 µM; lane 4, 11 - 0.4 µM; lane 5, 12 - 0.8 µM; lane 6, 13 - 1.0 µM; lane 7, 14 - 2.0 µM) were used for these assays. (**B**) Binding assays for the CENP–T–W–S–X complex to di-nucleosomes (0.1 µM) containing either CENP–A or histone H3 with 100 bp linker DNA. Different concentrations of the CENP–T–W–S–X complex (lane 2, 9 - 0.1 µM; lane 3, 10 - 0.2 µM; lane 4, 11 - 0.4 µM; lane 5, 12 - 0.8 µM; lane 6, 13 - 1.0 µM; lane 7, 14 - 2.0 µM) were used for these assays. (**C**) Visualization of the CENP–T–W–S–X-di-nucleosome complexes by an electron microscope (lower images). Images of di-nucleosomes in the absence of the CENP–T–W–S–X complex are also shown (upper images). Bar, 20 nm. (**D**) Competitive binding assays for the CENP–T–W–S–X complex to di-nucleosomes with 25 bp DNA and 100 bp DNA. Histone H3 nucleosomes were used in the left gel and CENP–A nucleosomes were used in the right gel. Different concentrations of the CENP–T–W–S–X complex (lane 4, 11 - 0.2 µM; lane 5, 12 - 0.4 µM; lane 6, 13 - 0.8 µM; lane 7, 14 - 1.0 µM) were used for these assays.

For these experiments, we prepared reconstituted di-nucleosomes containing either canonical human histone H3 or human CENP–A with a 25-bp linker DNA. We then mixed the chicken CENP–T–W–S–X complex with these di-nucleosomes and examined the mobility of the bound complexes by gel electrophoresis. As shown in Figure 2A, the CENP–T–W–S–X complex bound to both the H3–H3 and CENP–A–CENP–A di-nucleosomes. However, in both cases we did not observe a distinct shifted band. Instead, we observed larger complexes that were smeared in the gel, suggesting that multiple CENP–T–W–S–X complexes bind to a single di-nucleosome.

Based on this observed binding behavior, we hypothesized that the CENP–T–W–S–X complex does not associate with nucleosome-bound DNA in a regular manner, or that it may bind inappropriately to the 25-bp linker DNA and form larger complexes with di-nucleosomes through improper interactions (Figure 2A). As we have previously shown that the CENP–T–W–S–X requires ∼100 bp of naked DNA to bind in a regular fashion (12), we prepared di-nucleosomes with a 100-bp linker DNA and tested the interaction with the CENP–T–W–S–X complex. When we used either CENP–A or H3 di-nucleosomes with 100 bp linker DNA, the CENP–T–W–S–X complex bound to these nucleosomes forming a clear and distinct band (Figure 2B). This band is likely composed of a defined complex of the di-nucleosome and the CENP–T–W–S–X complex. Based on electron microscopy analysis, the di-nucleosome-containing DNA contains two globular complexes with a long-DNA stretch between them (Figure 2C, upper images). In contrast, when the CENP–T–W–S–X complex was added to di-nucleosome, we detected three globular complexes, indicating that the CENP–T–W–S–X bound to the 100-bp linker region (Figure 2C, lower images). The CENP–T–W–S–X complex bound to both the H3–H3 and CENP–A–CENP–A di-nucleosomes with 100 bp linker DNA with a similar binding efficiency (Figure 2B).

Finally, we conducted competition experiments to test whether the CENP–T–W–S–X complex bound preferentially to di-nucleosomes with a 100-bp linker relative to di-nucleosomes containing a 25-bp linker. As shown in Figure 2D, the competition experiments revealed that the CENP–T–W–S–X complex primarily bound to di-nucleosomes with 100 bp linker DNA, but not to di-nucleosomes with the 25-bp linker. Based on the combination of these binding experiments, we conclude that the CENP–T–W–S–X complex preferentially binds to ∼100 bp linker DNA rather than DNA present in nucleosomes.

### The CENP–T–W–S–X complex binds to a 100-bp DNA fragment primarily as a (CENP–T–W–S–X)_2_ structure

The CENP–T–W–S–X complex forms a heterotetramer in the absence of DNA (12). CENP–S and CENP–X also form a tetramer in the absence of CENP–T–W. Whereas the CENP–T–W–S–X complex localizes to kinetochores, the CENP–S–X complex associates with FANCM and is targeted to DNA damage sites in the absence of CENP–T–W (25,26). Despite the distinct function of these complexes, the structure of the CENP–T–W–S–X complex is similar to that of the CENP–S–X complex (12). However, we have previously shown that these complexes display distinct DNA binding properties (12). The CENP–S–X complex binds to DNA fragments of at least 40 bp, whereas the CENP–T–W–S–X complex bound preferentially to 100 bp DNA (Figure 2) (12).

**Figure 3.**
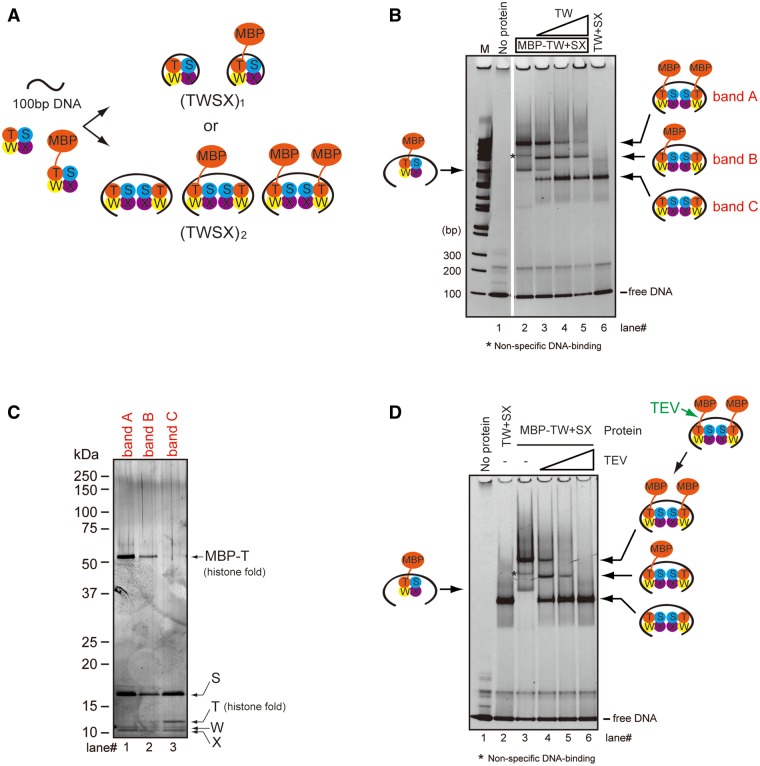
The CENP-T-W-S-X complex binds to 100 bp DNA as a (CENP-T-W-S-X)_2_ structure. (**A**) Experimental design to distinguish tetramer (TWSX)_1_ and two tetramers (TWSX)_2_ for binding to 100 bp DNA. Non-tagged CENP–T–W–S–X, MBP-fused CENP–T–W–S–X and the 100-bp DNA were prepared. If the CENP–T–W–S–X tetramer binds to 100 bp DNA, two bands would be detected. If the complex binds to 100 bp DNA as a (TWSX)_2_ form, three bands would be detected. (**B**) DNA-binding assays for the untagged CENP–T–W–S–X complex and the MBP-fused CENP–T–W–S–X complex to 100 bp DNA derived from pUC119. At final concentration of 1.25 µM, 100 bp DNA was used. The CENP–T–W complex (0.625 µM) or the MBP–CENP–T–W complex and the CENP–S–X complex (0.625 µM) were mixed and incubated. Different concentrations of the CENP–T–W complex (lane 3 - 0.313 µM; lane 4 - 0.625 µM; lane 5 - 1.25 µM) were added to the mixture of MBP–CENP–T–W and CENP–S–X complexes. The mixtures were analyzed by native PAGE. (**C**) Analysis of the protein composition of bands A, B, and C detected in (**B**). Proteins were extracted from each band and analyzed by SDS–PAGE. As expected, only MBP fused CENP–T was detected in band A, both MBP–CENP–T and non-tagged CENP–T were detected in band B, and only non-tagged CENP–T was detected in band C. We note that each subunit was not uniformly stained by the silver-staining method. We confirmed that we detected a similar staining profile by analyzing the purified CENP–T–W–S–X complex. (**D**) Cleavage assays for the complex of MBP-fused CENP–T–W–S–X (0.625 µM) with 100 bp DNA (1.25 µM) by TEV protease. Different units of TEV protease (lane 4 - 0.05 U; lane 5 - 0.1 U; lane 6 – 0.5 U) were used. Proteins after the TEV digestion were analyzed by native-PAGE. An asterisk indicates a non-specific band, as this band is invisible using different a DNA sequence (Supplementary Figure S1D).

Based on the tetrameric CENP–T–W–S–X structure and its predicted DNA-binding surface, we predicated that ∼60 bp of DNA would wrap around the CENP–T–W–S–X tetramer. However, as we observed that the CENP–T–W–S–X complex bound preferentially to a 100-bp DNA fragment, we hypothesized that a larger form of the CENP–T–W–S–X complex may bind to DNA. To test this, we prepared an MBP fusion to CENP–T to generate an MBP–CENP–T–W–S–X complex. We mixed the untagged CENP–T–W–S–X complex, the MBP–CENP–T–W–S–X complex, and a 100-bp DNA fragment and performed gel-shift assays. If the CENP–T–W–S–X complex binds to the 100-bp DNA fragment as a tetramer, two distinct bands for the untagged and MBP-tagged complexes should be visible. In contrast, if the CENP–T–W–S–X complex binds to DNA as two CENP–T–W–S–X tetramers, we should detect three bands corresponding to (MBP–CENP–T–W–S–X)_2_, (MBP–CENP–T–W–S–X/CENP–T–W–S–X) and (CENP–T–W–S–X)_2_, respectively (Figure 3A). Based on gel-shift assays, we found three major bands (bands A, B and C in lanes 3 and 4 of Figure 3B). To examine the protein composition of each band, we extracted proteins from the native-PAGE gel and separated them by SDS–PAGE. For the upper band (band A), we detected MBP–CENP–T, but not untagged CENP–T (Figure 3C, band A). Similarly, for the lower band (band C), we observed only untagged CENP–T, but not MBP–CENP–T (Figure 3C, band C). Importantly, we observed both MBP–CENP–T and untagged CENP–T in the middle band (band B) strongly suggesting that these proteins bind primarily to the 100-bp DNA fragment as two (CENP–T–W–S–X) tetramers (Figure 3C).

To confirm that the CENP–T–W–S–X complex binds to DNA primarily as a (CENP–T–W–S–X)_2_ structure, we next performed TEV protease cleavage using an MBP–CENP–T fusion with a TEV cleavage site between the MBP and CENP–T sequences. We combined MBP–CENP–T–W–S–X and the 100-bp DNA fragment to form a DNA–MBP–CENP–T–W–S–X complex and added TEV protease to the complex to remove the MBP tag. Prior to TEV protease addition, the upper band containing (MBP–CENP–T–W–S–X)_2_ was the major form (Figure 3D). However, following addition of limiting concentrations of TEV protease, we began to observe a middle band corresponding to (MBP–CENP–T–W–S–X/CENP–T–W–S–X) (Figure 3D). Finally, after addition of excess TEV protease, the band containing (CENP–T–W–S–X)_2_ became the major form and we did not detect MBP–CENP–T–W–S–X (Figure 3D).

In addition to the major (CENP–T–W–S–X)_2_ forms on DNA that we observed in the gel-shift assays, we also detected a minor lower band for the DNA–MBP–CENP–T–W–S–X (arrow, lane 2 of Figure 3B) and DNA–CENP–T–W–S–X (lane 4 of Supplementary Figure S1A and B) complexes. To clarify the nature of these bands, we compared their behavior to that of canonical octameric H3-containing nucleosomes bound to 146 bp DNA or the histone H3–H4 tetramer bound to 100 bp DNA. In these assays, the major band for the DNA–(CENP–T–W–S–X)_2_ complex (upper band in lane 4 of Supplementary Figure S1A) migrated at a similar position to the octameric histone nucleosome (lane 2 of Supplementary Figure S1A). In contrast, we found that the lower band for the CENP–T–W–S–X–DNA complex (lane 4 of Supplementary Figure S1A) migrated at similar position to the histone H3–H4 tetramer–DNA complex (lane 5 of Supplementary Figure S1A), suggesting that this corresponds to a tetrameric species. This lower CENP–T–W–S–X–DNA band was also migrated at similar position to the CENP–S–X–DNA complex, which corresponds to a tetrameric (CENP–S–X)_2_–DNA complex (Supplementary Figure S1C). Based on these data, we conclude that a minor potion of the CENP–T–W–S–X complex can bind to the 100-bp DNA as a tetramer, but that the complex primarily binds to the 100-bp DNA fragment as a (CENP–T–W–S–X)_2_ structure. We note that this conclusion is based on the migration of each protein–DNA complex in native PAGE, but that it is possible that this migration does not precisely reflect the molecular weight due to shape of the particles. In addition, although two CENP–T–W–S–X tetramers bind to the 100-bp DNA, in these assays we cannot distinguish whether two tetramers are located in tandem on DNA or whether the (CENP–T–W–S–X)_2_ structure is assembled into an octameric structure similar to the histones within a nucleosome. Crystallization of the complete DNA–(CENP–T–W–S–X)_2_ complex is an important next challenge to distinguish these possibilities.

### The CENP–T–W–S–X complex induces positive supercoils into DNA

We have shown previously that the CENP–T–W–S–X complex induces supercoils into DNA similar to canonical histones (12). For this experiment, we used a standard plasmid-supercoiling assay in which we detected a ladder of topoisomers that were induced by the CENP–T–W–S–X-wrapped DNA and that migrated faster than relaxed circular DNA. However, this assay did not test whether the direction of induced writhe of the supercoils was positive or negative. As there is an active debate on the topology of CENP–A-containing nucleosomes (14), it is important to define the topology of the CENP–T–W–S–X-containing nucleosome-like structure.

**Figure 4.**
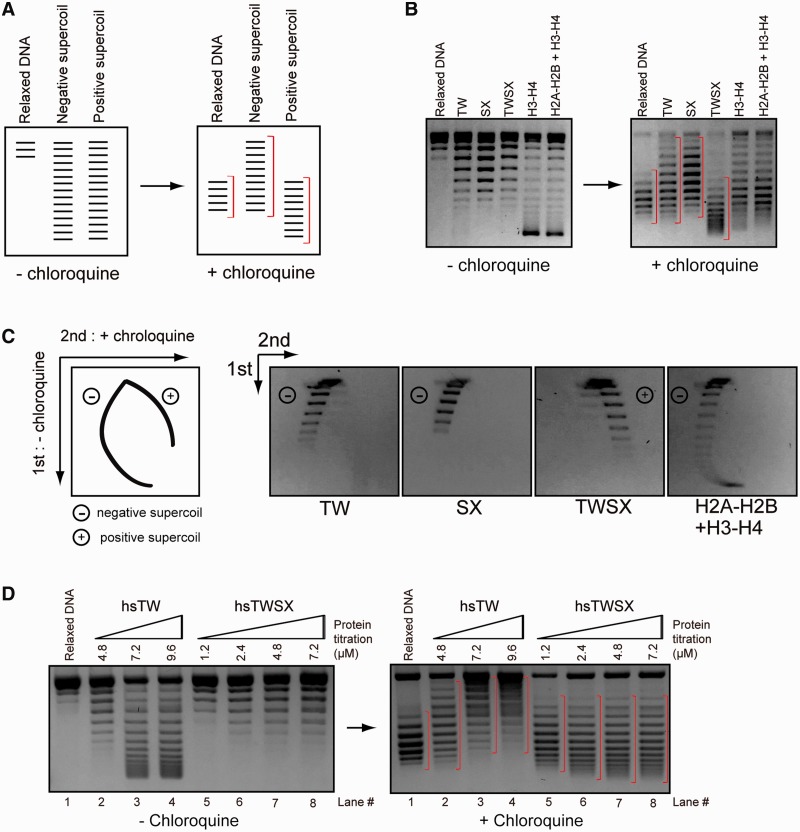
The CENP–T–W–S–X complex induces positive supercoils into DNA. (**A**) Experimental design to distinguish negative and positive supercoils. Negative supercoiled topoisomers slowly migrate following addition of chloroquine, whereas positive supercoiled topoisomers migrate faster following chloroquine addition. (**B**) Plasmid supercoiling assays performed with CENP–T–W, CENP–S–X, CENP–T–W–S–X, histone H3–H4 and histone H2A–H2B–H3–H4 complexes in the presence or absence of chloroquine. A pBluescript plasmid-containing chicken centromere DNA was used for these assays. (**C**) Two-dimensional gel electrophoresis of supercoiled toposiomers induced by CENP–T–W, CENP–S–X, CENP–T–W–S–X, histone H3–H4 and histone H2A–H2B–H3–H4 complexes. As shown in the diagram (left), electrophoresis was first performed in the absence of chloroquine and a second electrophoresis was performed in the presence of chloroquine, which clearly distinguishes positive and negative supercoils. The CENP–T–W–S–X complexes induced positive supercoils, whereas other complexes induced negative supercoils. A pBluescript plasmid-containing chicken centromere DNA was used for these analyses. (**D**) Plasmid supercoiling assays performed with human CENP–T–W and CENP–T–W–S–X complexes in the presence or absence of chloroquine.

To examine the direction of the supercoils induced by the CENP–T–W–S–X complex, we detected topoisomers by gel electrophoresis in the presence of the intercalating drug chloroquine (Figure 4A). Chloroquine causes negatively supercoiled topoisomers to migrate slowly, whereas positive supercoiled topoisomers migrate more quickly compared to the migration of relaxed DNA (Figure 4A). As a control, we used canonical H3-containing nucleosomes that have been shown previously to induce negative supercoils into DNA in this assay (15,16). Consistent with this, we found that histone octamers or the histone H3–H4 complex-induced negative supercoils into DNA (Figure 4B). Interestingly, whereas the CENP–T–W or CENP–S–X complexes alone induced negative supercoils into DNA similar to canonical histones, the CENP–T–W–S–X complex induced positive supercoils (Figure 4B and Supplementary Figure S2A).

To confirm these results, we analyzed the topoisomers using 2D gel electrophoresis in which samples were run in the absence of chloroquine in the first dimension and then run in the presence of chloroquine in the second dimension (see illustration in Figure 4C). Using this method, we could clearly distinguish positive or negative supercoils. In this assay, the CENP–T–W–S–X complex induced positive supercoils, whereas the CENP–T–W complex, CENP–S–X complex and canonical histone complexes induced negative supercoils (Figure 4C). We also observed similar results using the human CENP–T–W–S–X complex (Figure 4D). We found that the CENP–T–W–S–X complex-induced positive supercoils over a range of different salt concentrations (Supplementary Figure S2B and C) in contrast to archaeal histones that induce positive supercoils only at low salt concentrations (27). Finally, we found that the CENP–T–W–S–X complex-induced positive supercoils on a variety of different DNA templates including the plasmid pBluescript or ϕX174 DNA (Supplementary Figure S2D). Although we demonstrated that the CENP–T–W–S–X complex-induced positive supercoils into DNA, the supercoiling activity of the complex was weaker than that of canonical histone octamers as we detected an accumulation of topoisomers at the lowest position in a gel for histone octamer, whereas we did not detect topoisomers in this lowest position for the CENP–T–W–S–X complex (Figure 4B). Therefore, the CENP–T–W–S–X may not form regular nucleosome-like repeats on the plasmid DNA. In fact, when the supercoiled DNA induced by the CENP–T–W–S–X was partially digested with MNase, we observed a smeared DNA digestion pattern rather than a regular ladder as is observed for canonical nucleosomes (Supplementary Figure S2E), suggesting that the CENP–T–W–S–X complex binds to the plasmid DNA at random sites. However, based on the 2D electrophoresis, we emphasize that all supercoils induced by the CENP–T–W–S–X possess a positive writhe (Figure 4C).

In total, these results indicate that the CENP–T–W–S–X complex displays a distinct behavior on DNA relative to canonical histones with the ability to induce positive supercoils.

### The DNA-binding regions of CENP–T or CENP–W, but not CENP–S or CENP–X, are critical for the positive supercoiling activity of the CENP–T–W–S–X complex

**Figure 5.**
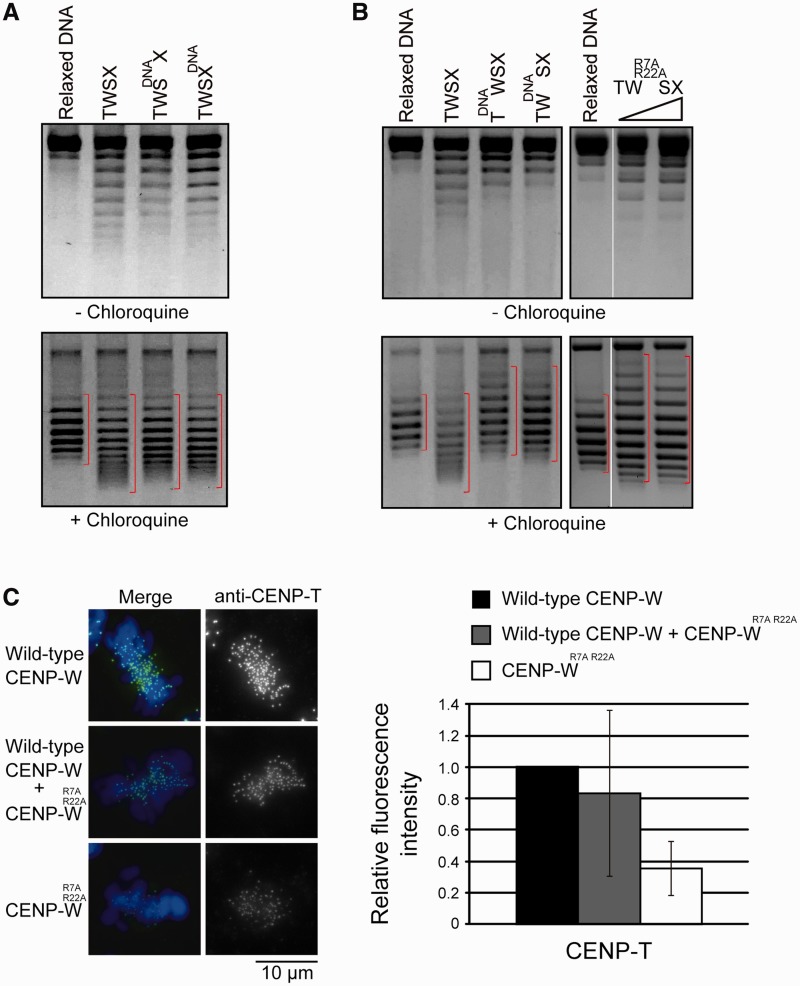
The DNA-binding sites of CENP–W are essential for the positive supercoiling activity of the CENP–T–W–S–X complex. (**A**) Plasmid supercoiling assays performed with the CENP–T–W–S^DNA^–X or the CENP–T–W–S–X^DNA^ complexes in the presence or absence of chloroquine. CENP–S^DNA^ and CENP–X^DNA^ mutants were prepared as described previously (12). (**B**) Plasmid supercoiling assays performed with the mutant CENP–T–W–S–X complex in the presence or absence of chloroquine. CENP–T^DNA^ mutant: Q543, R555 and K586 were replaced with A. CENP–W^DNA^ mutant: R7, R11, K12, R22, K54 and K56 were all replaced with A. CENP–W^R7A, R22A^ mutant: R7 and R22 were replaced with A. (**C**) Immunofluorescence of CENP–T in CENP–W–KO cells expressing either wild-type CENP–W, both wild-type CENP–W and mutant CENP–W^R7A, R22A^, or mutant CENP–W^R7A, R22A^. Signal intensities were measured in each cell line.

Finally, we analyzed the CENP–T–W–S–X complex to understand its DNA-binding properties and the positive supercoiling activity of the complex. Mutations in the DNA-binding regions of CENP–S or CENP–X [CENP–S^DNA^ or CENP–X^DNA^; (12)] did not affect the formation of positive supercoils into DNA (Figure 5A). In contrast, we found that mutations in the DNA-binding region of CENP–W (CENP–W^DNA^ or CENP–W^R7A, R22A^) or CENP–T (CENP–T^DNA^) prevented the CENP–T–W–S–X complex from inducing positive supercoils and instead induced negative supercoils (Figure 5B). For the CENP–W^R7A, R22A^ mutant, we found that the mutant complex did not bind properly to DNA and that a CENP–S–X tetramer formed following complex disassociation was primarily responsible for the observed DNA binding detected in these assays (Supplementary Figure S3). This suggests that the DNA-binding regions of CENP–T–W are critical for proper DNA binding and the positive supercoiling activity of the CENP–T–W–S–X complex.

The DNA-binding interface of CENP–T or CENP–W, but not CENP–S or CENP–X, is also critical for kinetochore targeting of the CENP–T–W–S–X complex (12). In fact, when we introduced CENP–W^R7A, R22A^ into CENP–W-deficient cells, we found that CENP–T localization was reduced by ∼70% compared to wild-type cells (Figure 5C). In summary, we conclude that CENP–T–W plays critical role for proper DNA binding and kinetochore targeting of the CENP–T–W–S–X complex.

## DISCUSSION

We have found that the CENP–T–W–S–X complex binds preferentially to 100-bp DNA fragments as a (CENP–T–W–S–X)_2_ structure, and induces positive supercoils into DNA. The formation of this higher order (CENP–T–W–S–X)_2_ structure may reflect two CENP–T–W–S–X tetramers located in tandem on DNA or an octameric structure similar to a nucleosome. Although we cannot distinguish both possibilities at present, the presence of two closely associated CENP–T molecules has important implications for understanding the mechanisms of kinetochore assembly. Our previous work has demonstrated that the N-terminal region of CENP–T extends outwards from the DNA to provide a platform for kinetochore assembly (10,22,23). CENP–T binds to the microtubule-binding Ndc80 complex in a stoichiometric manner. As the stable recruitment of the Ndc80 complex to kinetochores is essential for accurate chromosome segregation (1), its association with CENP–T is central to kinetochore function. The precise number of CENP–T molecules bound to DNA will define the number of Ndc80 molecules at kinetochores, directly influencing the behavior of the kinetochore–microtubule interface.

Although our work demonstrates that the CENP–T–W–S–X complex associates with DNA as a higher order histone-like structure, there are some intriguing differences between the CENP–T–W–S–X complex and a canonical histone octamer. First, histone octamers can be assembled under high salt conditions even in the absence of DNA, whereas a (CENP–T–W–S–X)_2_ form is not assembled under similar conditions, suggesting that two (CENP–T–W–S–X) tetramers do not tightly associate with each other in the absence of DNA. However, histone octamers are stabilized by DNA wrapping, and it is possible that assembly of an octameric CENP–T–W–S–X complex on DNA stabilizes this structure as well. Second, the CENP–T–W–S–X complex induces positive supercoils, whereas canonical nucleosomes induce negative coils into DNA. If two heterotetrameric (CENP–T–W–S–X) complexes are located side by side on DNA and each DNA-binding surface of the two (CENP–T–W–S–X) portions is located vertically on DNA, it would be possible for the (CENP–T–W–S–X)_2_ structure to induce a positive twist into DNA. Alternatively, if the CENP–T–W–S–X assembles into an octamer on DNA and DNA is wrapped in a right-handed direction, positive supercoils would also be formed. Although it is still unclear how the CENP–T–W–S–X complex induces positive supercoils, this feature is clearly distinct from canonical nucleosomes. Defining the structure of the DNA–(CENP–T–W–S–X)_2_ complex is an important next challenge to resolve these possibilities.

Unlike canonical nucleosomes, the CENP–T–W–S–X does not form regular nucleosome-like repeats on plasmid DNA *in vitro* (Supplementry Figure S2E). However, in cells other centromere proteins may be involved in the correct formation of the CENP–T–W–S–X-containing chromatin structure. Defining the nature of CENP–T–W–S–X-containing chromatin and its comparison with canonical nucleosomes are important questions for future work.

We propose that the CENP–T–W portion of the CENP–T–W–S–X complex is critical for the positive supercoiling activity of the CENP–T–W–S–X complex. In addition, the DNA-binding regions of CENP–T and CENP–W are essential for the kinetochore targeting of the CENP–T–W–S–X complex (12) (Figure 5). In contrast, CENP–T and CENP–W can be targeted into kinetochores in CENP–S-deficient cells or cells expressing CENP–S^DNA^ mutants (11,12), indicating that CENP–S and CENP–X are not essential for kinetochore targeting of the CENP–T–W–S–X complex. We found that mutations in the DNA-binding sites of CENP–S or CENP–W did not cause strong defects in the positive supercoiling activity of the CENP–T–W–S–X complex (Figure 5). Thus, CENP–S and CENP–X appear not to play significant roles for the targeting of the CENP–T–W–S–X complex to centromeres. However, CENP–T–W alone does not induce positive supercoils (Figure 4) and DNA-binding mutations of CENP–S cause defects in outer kinetochore assembly (12). Therefore, we conclude that both CENP–T–W and CENP–S–X contribute to proper kinetochore function and that the formation of the CENP–T–W–S–X complex is critical for the establishment of the centromere chromatin structure.

Although it is still unclear why centromere chromatin contains an opposite topology to canonical nucleosomes, one explanation is that the centromere must be distinct from other genome loci. Centromere regions are specified by sequence-independent epigenetic mechanisms, with CENP–A playing a key role as an epigenetic marker (2,4). However, there are likely to be additional features that are distinct from other genome loci. The positive supercoiling topology governed by the CENP–T–W–S–X complex may function to mark centromere regions.

## SUPPLEMENTARY DATA

Supplementary Data are available at NAR Online.

## FUNDING

A grant-in-Aid for Scientific Research (S) from the Ministry of Education, Culture, Sports, Science and Technology (MEXT), Japan (to T.F.); Cabinet Office, Government of Japan [Funding Program for Next Generation World-Leading Researchers to T.F.]; The International Human Frontier Science Program Organization (to T.N.); Grants-in-Aid for Scientific Research from MEXT (to T.N., T.H. and K.M.). PRESTO of JST (to T.H. and K.M.). Funding for open access charge: A grant-in-Aid for Scientific Research (S) from MEXT, Japan (to T.F.).

*Conflict of interest statement*. None declared.
